# Predicting Drug-Disease Associations via Using Gaussian Interaction Profile and Kernel-Based Autoencoder

**DOI:** 10.1155/2019/2426958

**Published:** 2019-08-27

**Authors:** Han-Jing Jiang, Yu-An Huang, Zhu-Hong You

**Affiliations:** ^1^Xinjiang Technical Institute of Physics and Chemistry, Chinese Academy of Science, Urumqi 830011, China; ^2^University of Chinese Academy of Sciences, Beijing 100049, China; ^3^Department of Computing, Hong Kong Polytechnic University, Hung Hom, Hong Kong

## Abstract

Computational drug repositioning, designed to identify new indications for existing drugs, significantly reduced the cost and time involved in drug development. Prediction of drug-disease associations is promising for drug repositioning. Recent years have witnessed an increasing number of machine learning-based methods for calculating drug repositioning. In this paper, a novel feature learning method based on Gaussian interaction profile kernel and autoencoder (GIPAE) is proposed for drug-disease association. In order to further reduce the computation cost, both batch normalization layer and the full-connected layer are introduced to reduce training complexity. The experimental results of 10-fold cross validation indicate that the proposed method achieves superior performance on Fdataset and Cdataset with the AUCs of 93.30% and 96.03%, respectively, which were higher than many previous computational models. To further assess the accuracy of GIPAE, we conducted case studies on two complex human diseases. The top 20 drugs predicted, 14 obesity-related drugs, and 11 drugs related to Alzheimer's disease were validated in the CTD database. The results of cross validation and case studies indicated that GIPAE is a reliable model for predicting drug-disease associations.

## 1. Introduction

For the past few decades, development of new drugs has been a time-consuming and costly process. The cost of developing new drugs has been rising in recent years, but the profits of new drugs are declining. Drug development is divided into three phases: the discovery phase, the clinical phase, and the clinical development phase. The first step in drug discovery phase is to identify potential drug-disease associations. Computational approaches for predicting drug-disease associations are drawing increasing attention in recent decades. Some redirected drugs have been successfully identified by rational observations. In view of the advantages of drug repositioning, it is an urgent need to utilize a more efficient approach for computational repositioning methods systematically.

In the past few years, a large number of computational methods have been proposed to predict drug-disease associations. For instance, Wang* et al.* proposed the TL-HGBI method, which is a computational framework based on a heterogeneous network model [1]. Martínez* et al.* built a network of interconnected drugs, proteins, and diseases and applied DrugNet to different types of tests for drug repositioning [2]. Lu* et al.* proposed a computational tool, DR2DI, to apply high-dimensional and heterogeneous omics data as information source to accurately reveal the potential associations between drugs and diseases [3]. Wu* et al. *developed a semisupervised graph cut algorithm, SSGC, to find the optimal graph cut [4]. Chen et al. used the recommendation system model to predict the relationship between drugs and diseases and put forward two different recommendation system models: ProbS and HeatS [5]. However, the data integration adopted by these methods only simply considers the linear combination between different types of features. Developing extensible and interpretive models by fusing multiple data sources remains challenging.

The drug repositioning problem is basically a prediction problem, which is usually defined as a classification task to solve. Classification tasks normally include two processes, feature extraction and classification. In the feature extraction process, the features of the drug and the features of the disease are, respectively, extracted, and then the drug and the disease feature are spliced into a drug-disease pair feature. In the process of classification, the extracted drug-disease features are classified by a classifier to obtain the classification results. For the approach of calculating features, the raw dataset as the materials of prediction problem has large noise information, which is the main challenge for the prediction task. Choosing a method for extracting the most meaningful features in each sample plays an important role in the subsequent prediction tasks. For example, Wang* et al.* used the fingerprints of the drug to generate Tanimoto score as the feature of the drug. But the features which they used only contained the structural information of the drug. Liang* et al.* combined the drug fingerprints to extract the characteristics of LRSSL and extracted important drug characteristics from multiple drug characteristic spectra under the constraint of L1-norm [6].

In recent years, in addition to the traditional feature extraction methods, feature extraction methods based on deep learning have been widely used. Autoencoder can learn features by reducing the dimension of the feature. For instance, Vishnubhotla* et al.* applied autoencoder to the modeling of low-dimensional coefficient model [7]. Badino* et al.* apply autoencoder for the unsupervised identification of subword units [8]. With the development of autoencoder and deep learning techniques, applications based on autoencoder have received more and more research attention. Using Autoencoder to map raw features to low-dimensional spaces can more effectively measure the relationship between drugs and disease. Along this promising direction, this work proposes a novel feature extraction method based on autoencoder for learning a meaningful feature representation of drug fingerprints. By doing so we can set objective function with respect to recovering new links on known drug-disease association network, considering the nonlinear combination of different features.

Computational methods based on statistical rules and machine learning can be used to supplement clinical trials to determine the relationship between drugs and disease at low cost. In addition, they can integrate different types of data resources related to diseases and drugs to generate experimental validation candidates. Much has been done in this promising direction. For example, SCMFDD applies similarity constrained matrix factorization to identify potential indications for a given drug [9]. LRSSL associates drugs with diseases through L1 regularized regression model, which improves the interpretability of the model [6]. SSGC is a semisupervised graph cut algorithm to find the optimal graph cut [4]. Inspired by the success of the recent machine learning approach, we have proposed a machine learning-based method to predict new drugs that are most likely to treat a particular disease.

Public databases store all drug-disease associations that have been confirmed by clinical studies, but a large number of unknown relationships remain to be studied. In this study, we propose a drug repositioning computational method combining Gaussian interaction profile kernel and autoencoder (GIPAE). GIPAE combines data information from multiple data sources, including drug Gaussian interaction profile kernel similarity, drug fingerprints, disease semantic similarity, and diseases Gaussian interaction profile. Secondly, a module based on autoencoder technology is built to extract the useful information of drug fingerprints and integrated the drug Gaussian interaction profile as the final drug feature descriptor. Similarly, disease Gaussian interaction profile similarity and semantic similarity are integrated into the final disease feature descriptor. Finally, the feature descriptor is used as the inputs of the random forest classifier to predict the association of each type of drug with all diseases. The purpose of our study is to establish an effective prediction model to look for new drug-disease association and to provide deeper understanding for the study of drug-disease association by looking for the influence factors.

To evaluate the performance of GIPAE, 10-fold cross validation was implemented on the Fdataset. GIPAE was compared with several state-of-the-art methods which were previously proposed for drug repositioning. The results show that the proposed method has better performance than the state-of-art methods. In addition, we validated the proposed model against two human disease including Obesity and Alzheimer disease. As a result, more than 10 of the top-20 drug candidates (14/20 for Obesity and 11/20 for Alzheimer disease) predicted by GIPAE were successfully confirmed by CTD database [10]. These experimental results indicated that GIPAE is effective to predict drug-disease associations on a large scale.

## 2. Materials and Methods

### 2.1. Datasets

In this work, we use two drug-disease association datasets following Gottlieb* et al.* and Luo* et al. *[11, 12]. As shown in Table 1, Gottlieb* et al.* collected 593 drugs, 313 diseases, and 1933 validated drug-disease associations from multiple data sources, which we here abbreviate as Fdataset. Luo* et al*. collected another dataset called Cdataset which covers 663 drugs, 409 diseases, and 2532 associations between them. The information of drugs is extracted from DrugBank, a comprehensive database containing extensive information about drugs [13]. The drug fingerprints defined in the PubChem database were extracted to represent the chemical substructures of drugs [14]. Disease information comes from human phenotypes definition in the Online Mendelian Inheritance in Man (OMIM) database, which focuses on human genes and disease [15]. In this work, we randomly generate negative samples from the unlabeled drug-disease pairs with the same number of the positive ones.

### 2.2. Feature Extraction Based on Autoencoder

**Figure 1:**
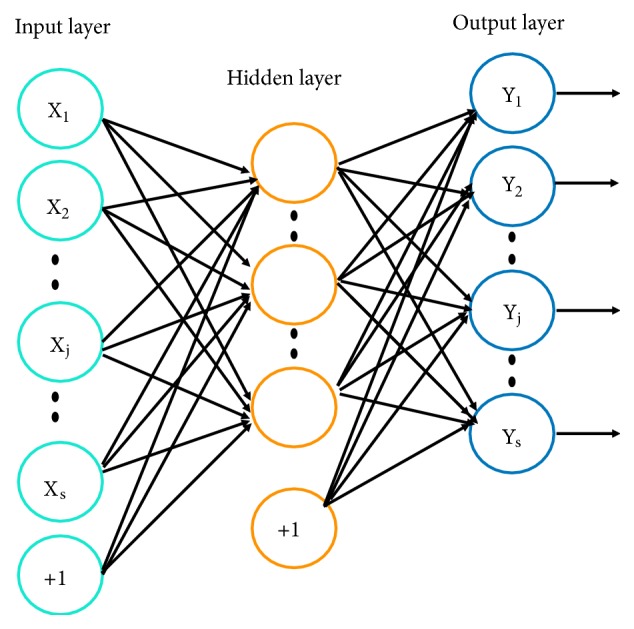
Structure of autoencoder.

Autoencoder has made remarkable progress with regard to its learning features for classification of more complex data such as image classification or voice recognition. It proves to be effective in solving different types of problems in data mining, gaining increasing attention for the application of deep learning-based applications in feature extraction for drugs. Autoencoder is a specific neural network structure, which is composed of two parts: encoder and decoder. As shown in Figure 1, it tries to learn a function:

(1) $$\begin{array}{c} \overset{´}{x}=f_{W,b}\left(\;x\;\right)\approx x \end{array}$$

where x is the input vector. W = (W_1_, W_2_) and b = (b_1_, b_2_) represent the variables for weights and the biases. Given a training sample set x, the autoencoder first encodes the input x into the hidden representation through a deterministic mapping as

(2) Y=σW1x+b1

(3) σx=11+exp⁡−x

*σ*(x) denotes an elementwise application of the logistic sigmoid. The resulting hidden representation, Y, is then mapped back to a reconstructed vector, $\overset{´}{\text{x}}$, with a similar mapping function:

(4) $$\begin{array}{c} \overset{´}{x}=\sigma \left(\;W_{2}Y+b_{2}\;\right) \end{array}$$

In this paper, the drug fingerprint is encoded and decoded by an autoencoder to obtain a matrix FG representing structural features.

### 2.3. Similarity for Drugs and Disease

The use of Gaussian interaction profile kernel can allow us to consider the nonlinear relationship of known drug-disease associations when we construct the feature representation. The method of Gaussian interaction profile kernel has been widely used in works relevant to disease prediction. For example, Chen et al. used Gaussian interaction profile kernel to calculate the similarity between MiRNA and disease when predicting Mirna-disease association [16]. Chen et al. used heterogeneous graphs to infer the association of Mirna-disease and used Gaussian interaction profile kernel to calculate the miRNA similarity and disease similarity [17]. Lu et al. used the Gaussian interaction profile kernel to calculate the disease similarity when predicting the drug-disease association [3]. The Gaussian interaction profile of the disease is calculated based on the assumption that similar diseases (e.g., different subtypes of lung cancer) can often bind to the same drug molecule and vice versa. The definition binary vector Y(d(x)) represents the interaction profiles of disease d(x) whose value describe whether d(x) is associated with each disease. The binary vector Y(d(x)) is equivalent to the x-th row vector of adjacency matrix. Then Gaussian interaction profile kernel similarity between d(x) and d(y) was defined as follows:

(5) GIPdisdx,dy=exp⁡−∂dYdx−Ydy2

where parameter ∂_d_ was implemented to tune the kernel bandwidth with normalizing original parameter as $\overset{´}{\partial_{d}}$ as follows:

(6) $$\begin{array}{c} \partial_{d}=\frac{\overset{´}{\partial_{d}}}{\left[\left(1/nd\right)\sum_{i=1}^{nd}{\left\|Y\left(d\left(x\right)\right)\right\|}^{2}\right]} \end{array}$$

Similarly, the definition binary vector Y(u(x)) represents the interaction profiles with drug u(x), and Y(u(y)) represents the interaction profiles with the drug u(y). Gaussian interaction profile kernel similarity for drug GIP_drug_ between u(x) and u(y) is calculated as follows:

(7) GIPdrug=exp⁡−∂uYux−Yuy2

(8) ∂u=∂u´1/nu∑i=1nuYux2

Here, the value of $\overset{´}{\partial_{u}}$ is set to 0.5 for simplicity, and the nu represents the number of the drugs.

We further calculate another type of disease similarity, that is, disease semantic similarity by using MimMiner, which measures disease similarity by calculating similarities between grid items. Specifically, we measure disease similarity using the similarity between MeSH terms and then compute similarity of correlation between drugs and diseases using known drug-disease association information. By applying the above method, the disease semantic similarity DS_r_ was obtained. We construct a new weighted disease sharing network based on the known drug-disease associations. The disease set represents the point of the network; the shared disease of the disease pair represents the weight. Diseases in the sharing network were clustered in groups by using ClusterONE [18]. ClusterONE is a method of graph clustering, which can be used to identify the cohesive modules in the weighted network. The cohesiveness of a cluster K could be defined by ClusterONE:

(9) $$\begin{array}{c} f\left(\;K\;\right)=\frac{C_{in}\left(K\right)}{\left(C_{in}\left(K\right)+C_{bound}\left(K\right)+P\left(K\right)\right)} \end{array}$$

where C_in_ represents the total weight of edges in vertex set K, *C*_*bound*_ represents the total weight of edges connecting the set with the rest of the group, and P is the penalty term. We assume that drug u_q_ and drug u_p_ are located in the same cluster K; the disease semantic similarity DS between drug u_q_ and drug u_p_ is defined as [12]

(10) $$\begin{array}{c} DS=\left(\;1+f\left(\;K\;\right)\;\right)\times {DS}_{r} \end{array}$$

In addition, for the disease semantic similarity between two diseases, if it is equal to or greater than 1, we use 0.99 instead.

### 2.4. Multisource Feature Fusion

In this study, we ultimately used descriptors that fused multiple sources of data including disease similarity, drug similarity, and drug fingerprint to predict the drug-disease association. There are some unknown associations for diseases/drugs in the dataset, and the corresponding Gaussian interaction profile kernel has a value of 0. To address this challenge, we use disease semantic similarity and drug structure similarity as a complement. The advantage of this method is that it can reflect the features of disease/drug from different perspectives.

We constructed two types of disease similarity, a semantic similarity model DS and a Gaussian interaction profile kernel similarity GIP_dis_. Calculate the disease similarity Sim(d(x), d(y))for disease d(x) and disease d(y) as

(11) $$\begin{array}{c} Sim\left(\;d\left(\;x\;\right),d\left(\;y\;\right)\;\right)=\left\{\begin{array}{cc} {GIP}_{dis}\left(d\left(x\right),d\left(y\right)\right) & if\;\;d\left(x\right)\;\;and\;\;d\left(y\right)\;\;has\;\;Gaussian\;\;interaction\;\;profile\;\;similarity\\ DS\left(d\left(x\right),d\left(y\right)\right) & otherwise \end{array}\right. \end{array}$$

We use a Gaussian interaction profile kernel similarity of a given disease pair (d(x), d(y)) to fill the feature matrix. If the disease Gaussian interaction profile kernel for a given disease pair (d(x), d(y)) is zero, then the disease semantic similarity is used to fill.

We integrate drug structure similarity FG and drug Gaussian interaction profile kernel similarity GIP_drug_ for the similarity of drugs. The formula calculating drug similarity RSim is

(12) $$\begin{array}{c} RSim\left(\;d\left(\;x\;\right),d\left(\;y\;\right)\;\right)=\left[\;{GIP}_{dis},FG\;\right] \end{array}$$

The RSim and FG feature matrices are spliced in line.

### 2.5. Feature Extraction in GIPAE

**Figure 2:**
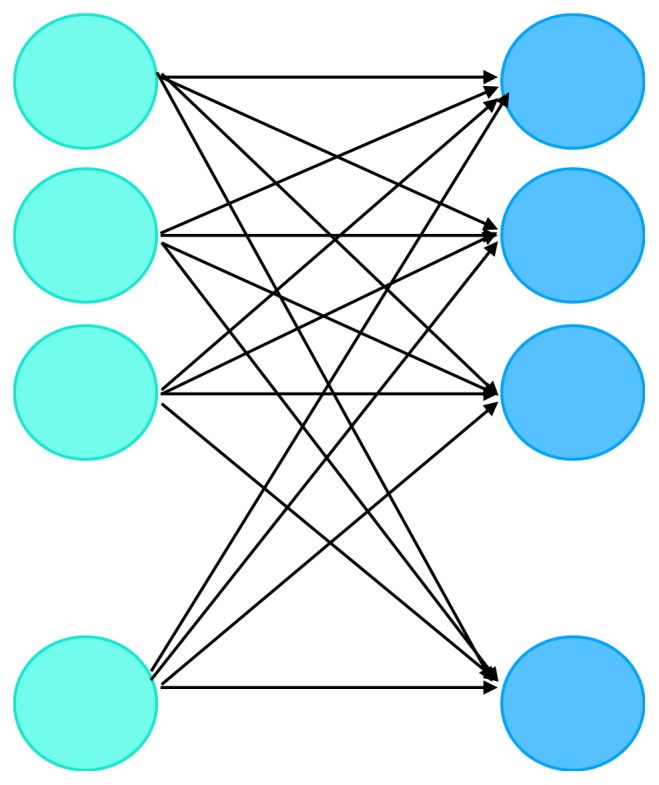
Structure of full-connected layer.

Deep learning has received extensive attention in the field of predicting drug-disease association. We introduce the batch normalization layer and the full-connected layer here to further improve the feature of drugs and disorders through deep learning. In the deep neural network training process, each batch sent to the network is usually trained in order, so that each batch has a different distribution. The Batch Normalization layer forcibly pulls the distribution of the neural network input values back to the standard normal distribution with a mean of 0 and a variance of 1. As shown in Figure 2, we introduce a full-connected layer to map features to the sample tag space. Here, the activation function of each neuron in the full-connection layer adopts ReLU function:

(13) $$\begin{array}{c} f\left(\;x\;\right)=\max\left(\;0,x\;\right) \end{array}$$

Ensemble learning algorithms have attracted increasing attention because they are more accurate than a single classifier. They are based on the premise that a group of classifiers is better than a single classifier. Random Forest (RF) has been widely applied in bioinformatics problems, including protein or peptide recognition, in vivo transcription factor binding prediction, and enhancer identification [19]. The RF consists of a combination of classifiers, each of which assigns the most frequent class to the input vector *ε* by a single vote.

(14) $$\begin{array}{c} \hat{C_{rf}^{B}}=majority\;\;vote{\left\{\;\hat{C_{b}}\left(\;\varepsilon \;\right)\;\right\}}_{1}^{B} \end{array}$$

where $\hat{C_{b}}\left(\varepsilon \right)$ is the class prediction of the b-th random tree. RF increases tree diversity by growing trees from different subsets of training data. Since RF corrects the habit of overfitting a training set by a decision tree, it generally has more stable prediction performance than other single classifiers such as SVM. In the GIPAE model, we chose random forests as our classifier.

**Figure 3:**
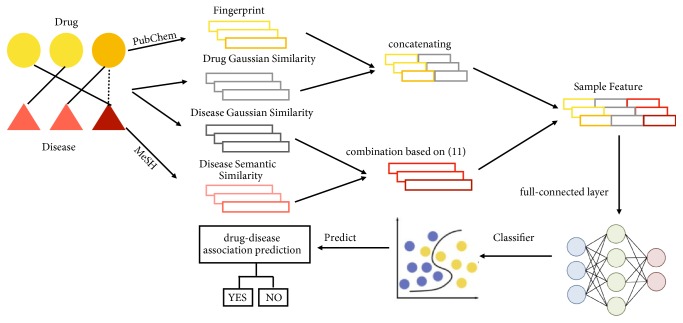
Flowchart of GIPAE model to predict potential drug-disease associations.

The process of feature representation consists of three steps (see Figure 3). Specifically, in the first step, the drug Gaussian interaction profile kernel is combined with drug fingerprint and disease Gaussian interaction profile kernel is combined with disease semantic similarity to obtain the drug and disease similar descriptors. The second step uses the full-connected layer to extract the features based on the combined drug and disease similarity. In its last step, a random forest classifier is introduced to yield the predicted scores using the outputs of second step as inputs.

## 3. Results and Discussion

### 3.1. Evaluation Criteria

To evaluate the performance of GIPAE, different types of evaluation criteria were used in this work to evaluate the performance of the proposed model, i.e., precision (Prec.), F1-score, Recall, and accuracy (Acc.).

(15) Prec.=TPTP+FP

(16) Recall=TPTP+FN

(17) F1-score=2TP2TP+FN+FP

(18) Acc.=TP+FNTP+TN+FP+FN

where TP, FP, and FN represent the number of positive samples correctly predicted in the model, denoting the number of correctly predicted negative samples, the number of falsely predicted positive samples, and the number of false predicted negative samples, respectively. For further evaluation, we also compute the receiver operating characteristic (ROC) curve, sum up the ROC curve in a numerical way, and calculate the area under the ROC curve (AUC).

### 3.2. Evaluate Prediction Performance

In this study, we trained machine learning models to predict whether certain drugs are related to certain diseases. The performance of the model was evaluated by cross validation method on Fdataset and Cdataset. In this method, all data sets are randomly divided into ten roughly equal parts for cross validation. Specifically, one group of them is taken as the test set, and the remaining nine groups are taken as the training set. Each time a different subset is used as the test set and the remaining nine subsets are used as training sets to form ten models. In this process, the Gaussian interaction profile kernel similarity uses the matrix obtained above. Finally, the ten models are used to predict the classification, and the average of ten models was taken as the final result.

We implemented our proposed method by using 10-fold cross validation on the Fdataset.  Table 2 shows that our proposed model yielded an average accuracy of 87.30%, precision of 86.06%, recall of 89.08%, and f1-score of 87.53% with standard deviations of 1.84%, 2.38%, 1.49%, and 1.74%, respectively.  Table 3 shows that, in the experiment on the Cdataset, our method yielded the average accuracy of 90.52%, precision of 89.77%, recall of 91.47%, and f1-score of 90.60% with standard deviations of 1.57%, 1.45%, 2.31%, and 1.61%, respectively.

**Figure 4:**
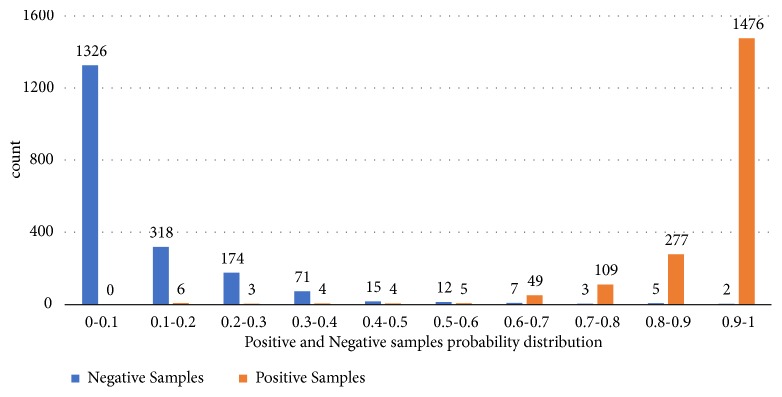
Positive and negative sample probability distribution.

We further statistically discuss the prediction result of 10-fold cross validation. As shown in Figure 4, we illustrate the distribution of predicted scores of positive and negative samples on Fdataset. As a result, for more than 85% of negative samples and 90% of positive samples, their predicted scores are lower than 0.2 and higher than 0.8, respectively. Specifically, 69% and 16% of negative samples' scores lie in the range of 0-0.1 and 0.1-0.2, respectively, while 76% and 14.3% of the positive samples achieve scores of 0.9-1.0 and 0.8-0.9, respectively.

These results indicate that the information including the Gaussian interaction profile kernel, the disease semantic similarity, and the drug fingerprint is sufficient to predict the interaction of a given drug-disease pair. The strong prediction performance of GIPAE model comes from the selection of drug-disease pair extraction method and machine learning classifier. Random forest classifier shows better performance due to ensemble model and random tree splitting strategy.

**Figure 5:**
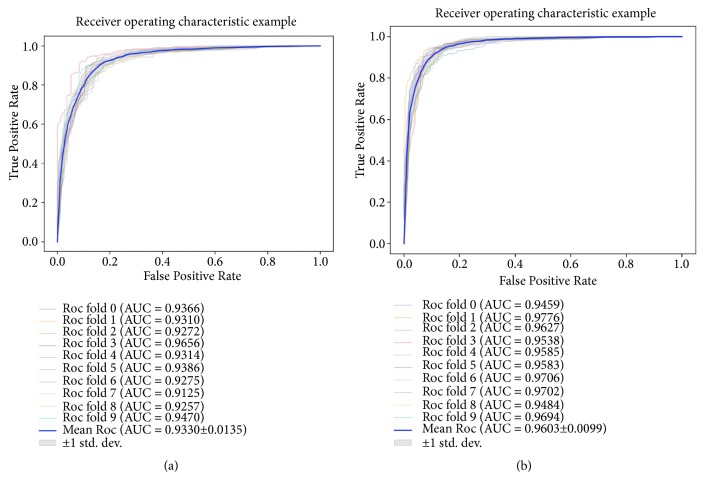
(a) and (b) Shown are the ROC curves yielded by GIPAE using 10-fold cross validation on Fdataset and Cdataset, respectively.

To further evaluate the performance of the proposed method, we compared it to the other five models previously proposed using the same ten-fold cross validation framework and exploring the same datasets. The five methods are MBiRW [12], DrugNet [2], HGBI [20], KBMF [21], and DRRs [22]. As shown in Figure 5 and Table 4, on Fdataset, our method achieves the highest AUC (Area Under the ROC Curve) which is 0.155 higher than that yielded by DrugNet, 0.104 higher than HGBI, and 0.018 higher than KBMF. MBiRW and DDRS yielded poor AUCs of 0.917 and 0.930. On Cdataset, GIPAE has an AUC of 0.960. DrugNet has an AUC of 0.804; MBiRW, HGBI, KBMF, and DRRS yielded AUC of 0.858, 0.928, 0.933, and 0.947. The results from both experiments demonstrate that the performance of GIPAE is significantly better than that of the other five models. Different from these comparison methods, our model has a wider application, using deep learning to express low-dimensional space, combined with ensemble classifier and random tree splitting strategy to achieve more significant prediction results.

### 3.3. Comparison among Different Classifiers

**Figure 6:**
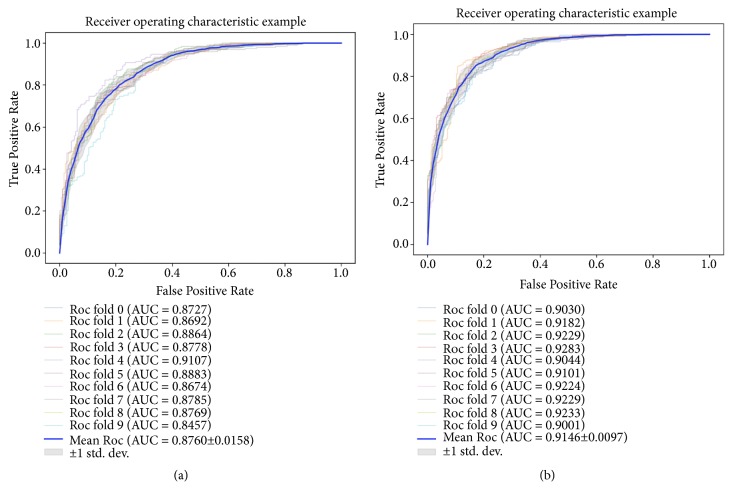
(a) and (b) Shown are the ROC curves yielded by SVM using 10-fold cross validation on Fdataset and Cdataset, respectively.

In this section, we try to replace random forest with Support vector machine (SVM) to compare the effectiveness of combination of the proposed feature extraction method and random forest classifier [23]. SVM is a widely used supervised learning algorithm with outstanding performance in classification and regression problems. Tables 5 and 6 show the results yielded by combining the proposed feature descriptor with support vector machine on Fdataset and Cdataset. On Fdataset, SVM achieved accuracy, precision, recall, and f1-score being 79.31%, 79.28%, 79.36%, and 79.30%. Their standard deviations are 1.58, 1.42, 2.69, and 1.75, respectively. On Cdataset, SVM achieved accuracy, precision, recall, and f1-score being 83.83%, 83.93%, 83.69%, and 83.80%, respectively. Their standard deviations are 1.60, 1.71, 1.92, and 1.62. It can be seen from the comparison that the classification result of the random forest classifier is better than the SVM classifier on F dataset and Cdataset. As shown in Figure 6, on Fdataset, the mean AUC is 0.8760. On Cdataset, the mean AUC was 0.9146. Among them, the average accuracy of random forests on Fdataset and Cdatasets is 7% and 6.69% higher than SVM, respectively. From the comparison we can see that, due to the ensemble model and its random tree splitting strategy, the random forest classifier can achieve better performance than the SVM classifier using the proposed feature descriptor.

## 4. Case Study

To further evaluate the predictive effect of this model, we selected Obesity and Alzheimer disease for case studies. Specifically, we use Fdataset as the training set to train the model. It is worth noting that when predicting the relevance of a particular disease, all associations between a particular disease and the drug should be removed. Take remaining drug-disease associations as positive sample. Then, among all unknown associations, the same number of associations with the positive sample is randomly selected as negative samples, and the negative samples are guaranteed not to be repeated. Based on the predicted results of GIPAE, the top 20 drugs were selected and compared with the CTD database.

Obesity is defined by the world health organization as abnormal or excessive accumulation of fat that poses a threat to human health. It is a major risk factor for many chronic diseases. As shown in Table 7, after we compared the predicted results with the CTD database records, 14 of the top 20 predicted drugs were confirmed. The other disease in our case study is Alzheimer disease, which is a neurodegenerative disease. Alzheimer disease is caused by a variety of factors, including biological and psychosocial factors.  Table 8 lists the top-20 drugs predicted by GIPAE to be associated with Alzheimer disease. Checking on CTD database, we successfully confirm 11 of them. It is worth noting that high-ranked interactions that have not been reported may also exist in reality. The case studies of Obesity and Alzheimer disease suggest that GIPAE has a good performance with regard to predicting the most promising diseases.

## 5. Conclusion

Drug-disease associations provide critical information for drug reposition. Biological experiments for drug repositioning are very expensive, so it is more advantageous to use computational methods to address drug repositioning problems. In this study, we proposed a novel computational method based on machine learning and deep learning called GIPAE. An underlying idea of the method that we propose is that the known drug-disease associations and drug fingerprint have a great influence on drug-disease associations. Specifically, GIPAE is a computational model based on Gaussian interaction profile kernel and autoencoder. It effectively integrates data on the association between known drugs and diseases.

We evaluated our proposed model on Fdataset and Cdataset datasets and tested them with 10-fold cross validation. On Fdataset, GIPAE obtained 86.06% prediction precision with 89.08% recall and the AUC of 93.30%. On Cdataset, GIPAE obtained 89.77% prediction precision with 91.47% recall and the AUC of 96.03%. These good experimental results show that our model can effectively predict the potential association between drugs and diseases. In addition, we conducted case studies on two complex human diseases (Obesity and Alzheimer disease) and found that more than half of the top 20 predicted results could be verified in the CTD database. These good experimental results indicate that this method can be used as a reliable application to predict potential interactions between drugs and diseases. In future work, we will consider ways to improve feature extraction to achieve higher prediction accuracy.

## Figures and Tables

**Table 1:** General statistics on Fdataset and Cdataset.

Datasets	Drugs	Diseases	Interactions
Cdataset	663	409	2532
Fdataset	593	313	1933

**Table 2:** Experimental results of 10-fold cross validation yielded by GIPAE on Fdataset.

Test set	Acc. (%)	Pre. (%)	Recall (%)	F1-score (%)
1	87.11	86.00	88.66	87.31
2	87.89	86.57	89.69	88.10
3	85.57	83.82	88.14	85.93
4	91.19	91.19	91.19	91.19
5	87.05	85.22	89.64	87.37
6	88.60	86.34	91.71	88.94
7	86.01	84.92	87.56	86.22
8	84.97	82.61	88.60	85.50
9	85.49	84.77	86.53	85.64
10	89.12	89.12	89.12	89.12
Average	*87.30±1.84*	*86.06±2.38*	*89.08±1.49*	*87.53±1.74*

**Table 3:** Experimental results of the 10-fold cross validation yielded by GIPAE on Cdataset.

Test set	Acc. (%)	Pre. (%)	Recall (%)	F1-score (%)
1	88.19	89.11	87.01	88.05
2	92.91	92.25	93.70	92.97
3	88.93	86.89	91.70	89.23
4	90.91	89.35	92.89	91.09
5	90.12	90.44	89.72	90.08
6	90.12	88.89	91.70	90.27
7	92.49	90.87	94.47	92.64
8	90.91	89.66	92.49	91.05
9	88.54	88.84	88.14	88.49
10	92.09	91.44	92.89	92.16
Average	*90.52±1.57*	*89.77±1.45*	*91.47±2.31*	*90.60±1.61*

**Table 4:** AUC results yielded by different methods using 10-fold cross validation.

	Fdataset	Cdataset
DrugNet [2]	0.778	0.804
HGBI [20]	0.829	0.858
KBMF [21]	0.915	0.928
MBiRW [12]	0.917	0.933
DRRS [22]	0.930	0.947
GIPAE (the proposed method)	0.933	0.960

**Table 5:** Results yielded by SVM on Fdataset using 10-fold cross validation.

Test set	Acc. (%)	Pre. (%)	Recall (%)	F1-score (%)
1	78.87	79.79	77.32	78.53
2	77.83	78.12	77.32	77.72
3	80.93	81.91	79.38	80.63
4	78.50	78.06	79.27	78.66
5	81.87	81.22	82.90	82.05
6	80.31	80.00	80.83	80.41
7	79.02	79.17	78.76	78.96
8	80.57	78.37	84.46	81.30
9	79.02	79.17	78.76	78.96
10	76.17	77.01	74.61	75.79
Average	*79.31±1.58*	*79.28±1.42*	*79.36±2.69*	*79.30±1.75*

**Table 6:** Results yielded by SVM on Cdataset using 10-fold cross validation.

Test set	Acc. (%)	Pre. (%)	Recall (%)	F1-score (%)
1	82.68	82.42	83.07	82.75
2	86.61	86.05	87.40	86.72
3	83.99	82.82	85.77	84.27
4	83.79	84.34	83.00	83.67
5	81.62	81.75	81.42	81.58
6	82.41	83.06	81.42	82.24
7	85.77	86.06	85.38	85.71
8	84.58	84.58	84.58	84.58
9	84.98	86.42	83.00	84.68
10	81.82	81.82	81.82	81.82
Average	*83.83±1.60*	*83.93±1.71*	*83.69±1.92*	*83.80±1.62*

**Table 7:** Top-20 drugs predicted by GIPAE to be associated with Obesity based on Fdatabase.

Index	Drug name	Evidence	Index	Drug name	Evidence
1	Phendimetrazine	Confirmed	11	Almotriptan	N.A.
2	Ticlopidine	Confirmed	12	Thioguanine	N.A.
3	Sibutramine	Confirmed	13	Terfenadine	Confirmed
4	Nitroglycerin	Confirmed	14	Sodium tetradecyl sulfate	N.A.
5	Mesalazine	Confirmed	15	Salsalate	Confirmed
6	Flutamide	Confirmed	16	Ramipril	N.A.
7	Felodipine	Confirmed	17	Procainamide	N.A.
8	Diethylpropion	Confirmed	18	Pravastatin	Confirmed
9	Desipramine	Confirmed	19	Phentermine	Confirmed
10	Captopril	N.A.	20	Orlistat	Confirmed

**Table 8:** Top-20 drugs predicted by GIPAE to be associated with Alzheimer disease based on Fdatabase.

Index	Drug name	Evidence	Index	Drug name	Evidence
1	Dopamine	Confirmed	11	Ranitidine	Confirmed
2	Methylergonovine	N.A.	12	Nizatidine	N.A.
3	Meperidine	N.A.	13	Nifedipine	Confirmed
4	Gemcitabine	Confirmed	14	Lithium	Confirmed
5	Betamethasone	Confirmed	15	Esomeprazole	N.A.
6	Pioglitazone	Confirmed	16	Ergocalciferol	Confirmed
7	Guanethidine	Confirmed	17	Dihydrocodeine	N.A.
8	Valrubicin	N.A.	18	Amitriptyline	Confirmed
9	Teriparatide	N.A.	19	Almotriptan	Confirmed
10	Rizatriptan	N.A.	20	Adenosine	N.A.

## Data Availability

The datasets that we collected in this work are freely available on https://github.com/HanJingJiang/GIPAE.
